# Autonomic aging – A dataset to quantify changes of cardiovascular autonomic function during healthy aging

**DOI:** 10.1038/s41597-022-01202-y

**Published:** 2022-03-23

**Authors:** Andy Schumann, Karl-Jürgen Bär

**Affiliations:** Lab for Autonomic Neuroscience, Imaging and Cognition (LANIC), Department of Psychosomatic Medicine and Psychotherapy, Jena University Hospital, Jena, Germany

**Keywords:** Risk factors, Ageing

## Abstract

Autonomic regulation of blood pressure and cardiac rhythm progressively declines with increasing age. Impaired cardiovascular control promotes a variety of age-related cardio-vascular conditions. This study aims to provide a database of high-resolution biological signals to describe the effect of healthy aging on cardiovascular regulation. Electrocardiogram and continuous non-invasive blood pressure signals were recorded simultaneously at rest in 1,121 healthy volunteers. With this database, we provide raw signals as well as basic demographic information such as gender and body mass index. To demonstrate validity of the acquired data, we present the well-known decline of heart rate variability with increasing age in this database.

## Abstract (summary)

**Table:** 

Measurement(s)	Cardiovascular autonomic function
Technology Type(s)	Physiological signals
Sample Characteristic - Organism	Homo sapiens

## Background & Summary

As the elderly population has a high prevalence of cardiovascular diseases, representing the globally leading cause of death, it is essential to detect bodily changes that adumbrate cardiovascular impairments^1^. In addition, impairment of autonomic regulation of the cardiovascular system seems to accelerate the decline of cognitive performance and promotes the risk to develop age-related diseases^2,3^.

It is well established that arterial and ventricular stiffening, reduction of myocardial contractility, or degeneration of organ innervation impair cardiovascular function in an aged population and promote sustained hypertension, thrombosis, and atherosclerosis^4^. Recent scientific studies have suggested that an elevation of blood pressure and signs of thrombosis in midlife increase the risk to develop Alzheimer’s disease or other dementias in later life^5,6^.

A decreased sensitivity of vagal reflexes such as respiratory sinus arrhythmia leads to elevated heart rates and diminished heart rate variability in elderly participants^7–9^. The feedback loop decelerating heart rate due to increasing blood pressure, i.e. baroreflex function, is progressively diminished with increasing age. Structural changes such as the loss of sinoatrial pacemaker cells and left ventricular hypertrophy as well as functional changes of receptors initiating autonomic reflexes (e.g. baroreceptors) contribute to an altered cardiovascular regulation related to aging^10,11^.

Lower levels of vagally modulated HRV have been associated with increased cardiovascular morbidity and mortality in the elderly^12^. Hillebrand *et al*. reported that healthy participants with diminished resting heart rate variability (HRV) have a 32–45% increased risk to suffer from a first cardiovascular event^13^. Resting heart rate and HRV were demonstrated to predict cardiovascular disease as well as cardiac and overall mortality^14^.

In this study, we enrolled healthy volunteers between 2010 to 2020. Participants were recruited from the local community as well as employees of the University Hospital. We recorded electrocardiograms and continuous noninvasive blood pressure at carefully controlled resting state conditions in our laboratory with a constant well-defined room temperature and humidity, a defined illumination level and without any environmental noise. Available data might be used to describe the influence of aging on cardiovascular autonomic regulation to better understand mechanisms of healthy aging in contrast to pathological changes.

## Methods

### Participants

Resting state physiological recordings of 1,121 healthy volunteers were obtained. Exclusion criteria were any medical conditions, illegal drugs or medication potentially influencing cardiovascular function. Thorough physical examination, resting electrocardiography (ECG) and routine laboratory parameters (electrolytes, basic metabolic panel, and blood count) had to be without any pathological finding (results are not part of the database). Demographic information is depicted in Table 1.

**Table 1:** Demographic information on the entire sample of healthy volunteers and divided into subgroups that have been recorded with one of the two measurement systems (TFM: CNSystems Task Force Monitor and BIOPAC MP150 with CNSystems CNAP 500).

Parameter	Total (N = 1,121)		TFM (N = 621)		MP150 (N = 500)	
	Mean ± SD	Missing	Mean ± SD	Missing	Mean ± SD	Missing
Age [years]	32.5 ± 14.7	25	33.5 ± 15.7	24	31.3 ± 13.3	1
Gender [f/m]	670/433	2	383/243	0	287/190	2
BMI [kg/m²]	23.7 ± 4.5	0	23.9 ± 5.0	0	23.4 ± 3.6	0

BMI: body mass index. SD: standard deviation.

### Human subjects

The study was approved by the ethics committee of the Friedrich-Schiller-Universität Jena. All research was performed in accordance with relevant guidelines and regulations. Informed written consent and consent for anonymous data sharing were obtained from all participants.

### Procedures

All measurements were recorded at the Department of Psychosomatic Medicine and Psychotherapy at Jena University Hospital.

### ECG

**Fig. 1:**
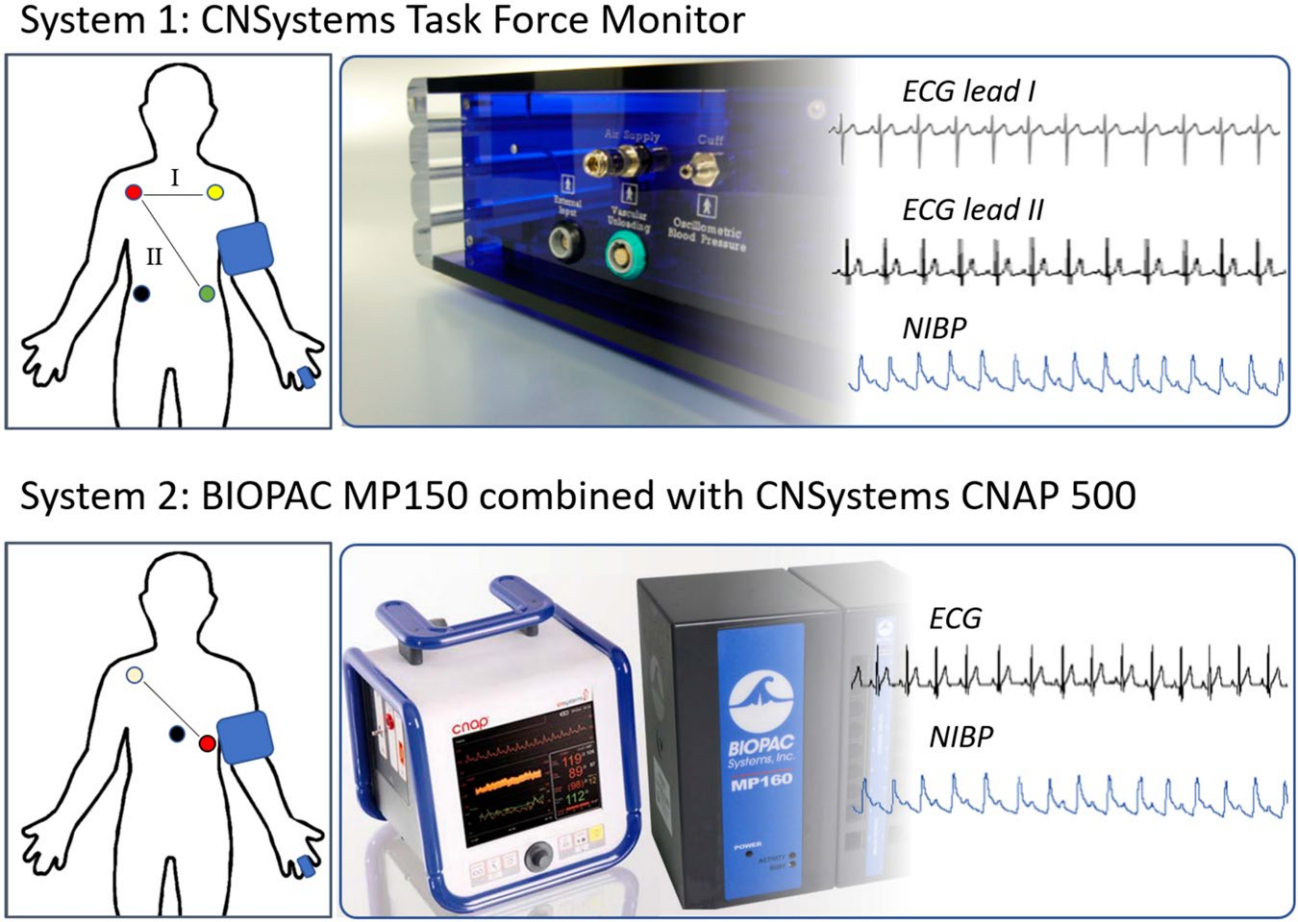
Cardiovascular recordings using system 1 - the Task Force Monitor (CNSystems), or system 2 – the CNAP 500 (CNSystems) combined with an MP150 acquisition unit (BIOPAC Systems Inc.). Top left: In System 1, four electrodes have been attached at the right arm (red), the left arm (yellow), the left abdomen (green), and the right abdomen (black). Two ECG channels have been recorded between the electrodes red and yellow (lead I), and between red and green (lead II). Pressure cuffs have been attached around the left arm as well as left middle and index finger. Top right: Two ECG leads were sampled synchronously at 1000 Hz and the continuous blood pressure signal at 100 Hz. Bottom left: In System 2, three electrodes have been attached below the centre of the right clavicle (white), at the left lower thorax (7th intracoastal space at midclavicular line) (red), and at the sternum (black). One ECG channel has been recorded between the electrodes red and white (lead II). Pressure cuffs were attached around the left arm as well as left middle and index finger. Bottom right: The ECG was sampled synchronously at 1000 Hz and the continuous blood pressure signal at 100 Hz. Pictures from www.cnsystems.com and www.biopac.com adapted with permission.

An ECG (lead II) was recorded at 1000 Hz either by an MP150 (ECG100C, BIOPAC systems inc., Golata, CA, USA) or Task Force Monitor system (CNSystems Medizintechnik GmbH, Graz, AUT). Pre-gelled Ag/AgCl electrodes (BlueSensor VL, Ambu GmbH, Bad Nauheim, GER) were attached according to an Einthoven triangle (Fig. 1). ECG and continuous blood pressure were digitalized synchronously by the analog-to-digital converter incorporated in the main system (MP150 or Task Force Monitor).

### Blood pressure

Continuous blood pressure was recorded non-invasively using the vascular unloading technique^15^. In short, a cuff around the finger is controlled to maintain constant pressure, while blood volume is recorded via photoplethysmography. With non-varying cuff pressure, the acquired blood volume is proportional to blood pressure in the arteries of the finger. The recorded signal is calibrated by an oscillometric measurement of the brachial blood pressure once during initialization of the system (measurement accuracy ± 3 mmHg). The Task Force Monitor is equipped with a module for continuous blood pressure measurement. The MP150 system digitizes the signal acquired by a separate monitor CNAP 500 (CNSystems Medizintechnik GmbH, Graz, AUT). The sampling frequency was 100 Hz for both systems.

### Missing data

During some recordings, the system measured brachial blood pressure again for re-calibration. This led to loss of continuous NIBP signal over a short time span. Missing data points have been set to *NaN*. When demographic data was missing, respective cells were filled with *NaN*.

### Parameters

To demonstrate plausibility of collected data, we investigated a number of fundamental heart rate variability indices and their dependence on age. Therefore, heart beats were extracted and analyzed using the freely available PhysioNet Cardiovascular Signal Toolbox implemented in MATLAB (R2019a, The Mathworks, Natick, MA, USA). As exemplification, standard deviation of heart beat intervals (SDNN), root mean squared difference of successive heart beat intervals (RMSSD), and deceleration capacity (DC) was assessed using the PhysioNet Cardiovascular Signal Toolbox^16^. For pre-processing of heart beat interval time series, we used an adaptive filter algorithm that is also available online^17^. Outliers in age bins were excluded when exceeding three times the median absolute deviation^18^.

### Measurement protocol

Measurements were performed in our autonomic laboratory that was temperature controlled at 22 °C. During recordings, the environment was quiet and fully shaded. The illumination level was kept constant via an indirect light source. The recording session started with an interview of the participant. Then, the purpose and design of the study was explained. All study volunteers gave his or her written consent to participate. Weight was measured using a person scale (SECA GmbH & Co. KG., Hamburg, GER) and height using a stadiometer (Harpenden, Holtain Ltd., Crosswell, UK).

After participants lied down comfortably on the examination tilt table, electrodes and pressure cuffs were placed. For the resting state recording, participants were instructed to avoid movement, yawning or coughing.

The instructor waited a few minutes for the participant to calm down and checked the quality of the acquired signals. In case of insufficient signal quality, electrodes and cuffs were re-arranged. Otherwise, the recording was started. The length of the recording was 8 to 35 minutes and was supervised by the instructor.

## Data Records

Data are available at PhysioNet^19,20^. Each recording is stored as a data file with signals of ECG and blood pressure. Blood pressure values were spline-interpolated and resampled to 1000 Hz. Additional information can be found in *subject-info.csv* that is included in the repository.

### Data anonymization

Data was anonymized using the free open-source Data Anonymization Tool ARX^21^. To assure that none of our participants can be identified based on demographic information, we generalized individual age to age groups. The applied a k = 2 anonymity condition and an average re-identification risk of 5%^22,23^. Files were pseudonymized to numbers 0001–1121 after random ordering.

Recorded data of each participant are stored in a data file (.dat) with raw signals of ECG and blood pressure. Amplitudes are included as a sequence of unsigned 16-bit integers for each signal. An associated header file is an ASCII text file storing meta information on this recording. The first line gives file name, number of signals, sampling frequency and number of samples separated by spaces (e.g., first line in *0023.hae*: ‘0023 2 1000 1226975’). Each of the following lines include information regarding one signal, namely, file name, defined bit resolution, bit gain, baseline in brackets followed by a slash, units, bit resolution followed by a zero, first bit, checksum followed by a zero and signal name (e.g., second line in *0023.hae*: ‘0023.dat 16 10688.4581(−12269)/mV 16 0 −13073 17059 0 ECG’). There is a script to convert wfdb files to mat format (and reverse) in the Waveform Database Software Package for MATLAB (*wfdb2mat.m*) available at PhysioNet^24^.

The table *subjects.tsv* gives basic demographic information i.e., age bin, gender and body mass index as well as length of recording in minutes and recording device.

## Technical Validation

**Fig. 2:**
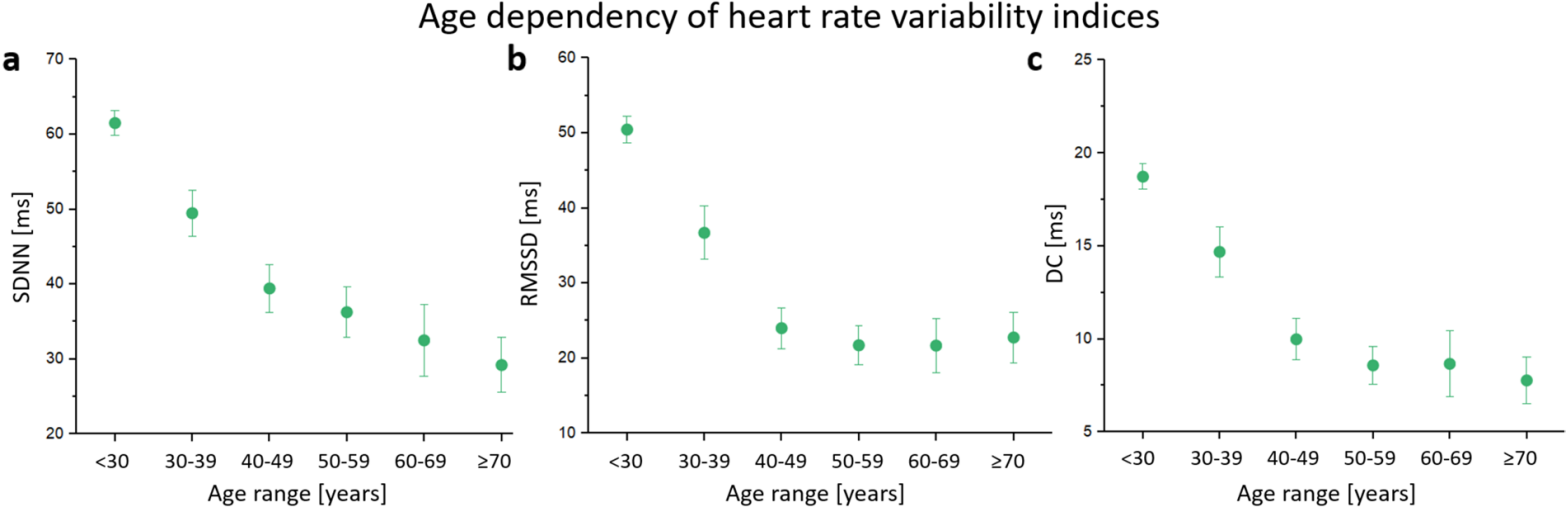
Age dependency of heart rate variability. (**a**) Standard deviation of heart beat intervals in age bins (SDNN). (**b**) Root mean square of successive differences between heart beat intervals in age bins (RMSSD). (**c**) Deceleration capacity of heart beat intervals in age bins (DC).

To explore validity of recorded data, the sample was divided into age bins of ten years (see Fig. 2). We estimated standard deviation of heart beat intervals (SDNN) and root mean square of successive differences between heart beat intervals (RMSSD), and deceleration capacity of heart beat intervals. Global heart rate variability as indicated by SDNN seems to decline progressively with advancing age (Fig. 2). DC and RMSSD suggested that short-term modulation of heart rate decreases until about 50 years of age in a linear matter. At a higher age both parameters remain nearly constant.

## Data Availability

For the technical validation, we used code that is publicly available without restrictions (see methods section). In detail following functions/scripts were used: *jqrs.m* - QRS detector based on the Pan-Tompkins method (www.physionet.org/content/pcst) *ada_f.m* – adaptive filtering of heart beat time series (www.tocsy.pik-potsdam.de/ada.php) *runrmssd.m* – RMSSD estimation (www.physionet.org/content/pcst) *prsa.m* – deceleration capacity estimation (www.physionet.org/content/pcst) *wfdb2mat.m –* convertion of waveform database format to MATLAB file (www.physionet.org/content/wfdb-matlab/0.10.0/mcode/wfdb2mat.m).
